# LncRNA MALAT1 promotes high glucose-induced inflammatory response of microglial cells via provoking MyD88/IRAK1/TRAF6 signaling

**DOI:** 10.1038/s41598-018-26421-5

**Published:** 2018-05-29

**Authors:** Li-Qing Wang, Heng-Jun Zhou

**Affiliations:** 1 Department of Anesthesiology, The First Affiliated Hospital of Zhejiang University, Hangzhou, 310003 China; 2 Department of Neurosurgery, The First Affiliated Hospital of Zhejiang University, Hangzhou, 310003 China

## Abstract

Although a large number of studies have confirmed from multiple levels that diabetes mellitus (DM) promotes cerebral ischemic reperfusion (I/R) injury, but the precise mechanism is still unclear. A cerebral I/R injury model in diabetic rats was established. The neurological deficit scores and brain edema were monitored at 24 and 72 hours after injury. The peri-infarct cortical tissues of rats were isolated for molecular biology detection. The rat primary microglia and microglia line HAPI were cultured to establish the cell model of DM-I/R by high glucose (HG) and hypoxia-reoxygenation (H/R). The endogenous expression of MALAT1 and MyD88 was regulated by the transfection with pcDNA-MALAT1, si-MALAT1 and si-MyD88, respectively. The cerebral I/R injury model in diabetic rats had more severe neuronal injury as shown by the significantly higher neurological deficit scores and an obvious increasing brain edema at 24 and 72 hours after injury. Moreover, the microglia were activated and induced a large number of inflammatory cytokines TNF-α, IL-1β and IL-6 in the peri-infarct cortical tissues during cerebral I/R injury associated with DM. The expression of MALAT1, MyD88, IRAK1 and TRAF6 protein were significantly up-regulated by DM-I/R *in vitro* and *in vivo*. Furthermore, the HG-H/R-induced MALAT1 promoted the inflammatory response in microglia via MyD88/IRAK1/TRAF6 signaling. Our results suggested that MALAT1 mediated the exacerbation of cerebral I/R injury induced by DM through triggering the inflammatory response in microglia via MyD88 signaling.

## Introduction

Cerebrovascular disease has the characteristics of high incidence, disability rate and mortality rate that seriously damage the health of human^1^. Diabetes mellitus (DM) is a metabolic disease characterized by high blood sugar, the serious harm to human health. As an independent risk factor for cerebrovascular diseases, DM can induce and aggravate ischemic cerebrovascular diseases, which leads to cranial nerve injuries or die^2–4^. It was found that DM promoted the deterioration of cerebral ischemia and led to a great deal of apoptosis of nerve cells in ischemic penumbral zone (IPZ), as well as in brain midline areas^5^. IPZ, damage areas, and necrosis areas in the brain of diabetic patients were significantly greater than that of nondiabetic patients^6^. Although a large number of studies have confirmed from multiple levels that DM promoted cerebral ischemic reperfusion injury, but the precise mechanism is still unclear.

Metastasis associated lung adenocarcinoma transcript 1 (MALAT1), a long non-protein coding RNA (lncRNA), plays a major role in a variety of pathological and physiological circumstances. MALAT1 was originally confirmed to be involved in the development and metastasis of tumour^7^. Subsequently, MALAT1 was shown to participate in diabetic retinopathy (DR) by regulating inflammatory response^8^. Recently, it was found that MALAT1 mediated the glucose-induced inflammatory cytokine production, including tumour necrosis factor alpha (TNF-α) and interleukin 6 (IL-6), in the endothelial cells, which may lead to the development of DM-induced vascular complications^9^. Furthermore, MALAT1 was dramatically increased in the kidneys of diabetic mice accompanied by a relatively high level in IL-6 and TNF-α mRNA^9^. Although these findings suggest that MALAT1 is closely relates to the DM-induced complications, the involvement of MALAT1 in DM-associated cerebral ischemic reperfusion injury is not yet known.

The inflammatory reaction is implicated in the occurrence of DM-associated cerebral ischemic reperfusion injury^10,11^. The inflammatory response of the central nervous system (CNS) is mainly characterized by the activation of microglia and astrocytes^12^. And the excessive activation of microglia induced a large number of neurotoxic substances and proinflammatory cytokines, further aggravating the diabetic cerebral infarction injury^13,14^. Thus, the regulation of microglia activation may be one of the effective interventions for ischemic brain injury.

Myeloid differentiation factor-88 adaptor protein (MyD88) mediated the activation of interleukin-1 receptor (IL-1R) signaling pathway and NF-κB to induce inflammatory cytokines such as TNF-α^15^. IL-1R is expresses not only in immune cells but also in microglia^16^. Moreover, MyD88 signaling promoted the inflammatory responses induced by cerebral ischemic reperfusion in murine models^17^. In the present study, we hypothesized that lncRNA MALAT1 participated in the pathogenesis of the cerebral ischemic reperfusion injury induced by DM, the mechanism of which may be related to the MyD88-mediated inflammatory response in microglia.

## Methods

### Animals

Forty-eight healthy Sprague-Dawley (SD) rats (sex: male, weight: 210–230 g, age: 6–8 weeks) were purchased from Shanghai Laboratory Animal Center (SLAC; Shanghai, China). SD rats were raised in the individual cages with the standard laboratory rearing temperature held at 23–25 °C and free food and water that were offered under the 12 h cycle of light and dark. All SD rats were suffered from acclimatization for a week before treatment. Experiments performed on animals obtained the approval of the experimental animal ethical committee of the First Affiliated Hospital of Zhejiang University. We confirmed that all methods were performed in accordance with the relevant guidelines and regulations.

### The establishment of animal models

The rats were divided into four groups at random: Control-sham group (n = 6), Control-I/R group (n = 6), DM-sham group (n = 6) and DM-I/R group (n = 6). To establish the DM model, the SD rats were intraperitoneally injected with streptozotocin (STZ; Sigma-Aldrich, MO, USA) dissolved in 0.1 mol/l citrate buffer at the dose of 60 mg/kg. After 72 h, the serum glucose level in rats was more than 16.7 mmol/L, which indicated that establishment of DM model was successful.

The rats in Control-sham group and Control-I/R group were received the injection of 0.1 mol/L citrate buffer. The rats of DM-I/R group and Control-I/R group were anesthetized using chloral hydrate (350 mg/kg, i.p.), followed by performed to establish I/R model using middle cerebral artery occlusion (MCAO) method^18^. After anesthetized, the rats were exposed to the left common carotid artery, internal carotid artery (ICA) and external carotid artery (ECA). Whereafter, the latter of ECA were ligated while the branch vessels were blocked. The monofilament nylon suture thread with a length of 18–20 mm and a diameter of 0.24–0.26 mm was inserted into the right ICA via the ECA until a slight resistance was obtained while the CCA and ECA were blocked by clips. The suture was left in place for 2 h and removed to the reperfusion. The rats of the other two groups were modeled with the same procedure as above but not inserting the suture thread.

At 24 h or 72 h after reperfusion, neurological deficit score of each rat was evaluated according to the criteria of Longa 5 scores^19^. The neurological deficit of rats was graded as follows: 0 score, normal walk without any neurological symptoms; 1 score, impaired in extending contralateral forelimb; 2 scores, circling toward the contralateral side; 3 scores, fall toward the contralateral side; 4 scores, impaired in walk and unconsciousness, most severe neurological deficit. And the brain tissues were isolated from the killed rats, used for the next experiment. In addition, the brain tissues was cut at 2 mm consecutively afer being frozen at −20 °C for 15 mins in the cryostat.

### Volume of encephaledema

The 2,3,5-triphenyl-2H-tetrazolium chloride (TTC) (2%) solution was used to strain the brain slices at 37 °C for 30 mins in the dark chamber, followed by that 4% polyformaldehyde was used to fix the brain slices for 24 h. AUTOCAD2000 (Autodesk) was used to analyze the images of the brain slices. The contralateral and ipsilateral hemispheres of the ischemia brain were presented as V1 and V2, respectively. The volume of cerebral edema was equal to V1 minus V2 (mm^3^).

### Enzyme-linked immunosorbent assay (ELISA)

The concentrations of IL-1β, IL-6 and TNF-α in the brain tissue were measured using specifc ELISA kits following the instructions of the manufacturer (ShengGong Biological Technology, Shanghai, China). For detection of IL-1β, IL-6 and TNF-α in the ischemia brain tissue, the tissue was homogenized on ice to collect the supernatant by centrifugation at 2,500 × g for 20 mins. The amounts of IL-1β, IL-6 and TNF-α were detected using ELISA kits with an ELISA reader (Bio-Rad Laboratories, Richmond, CA) at 450 nm. Each experiment was repeated three times.

### The measurement of MALAT1, Emr1, CD68, IL-1β, IL-6 and TNF-α

Trizol reagent (Invitrogen) was used to extract the total RNA. Then the reverse transcription reactions were performed with the PrimeScript RT Enzyme mix kit (Takara) to obtain the cDNA for the next reaction. The synthesized cDNA was used with Fast SYBR Green PCR kit (Applied Biosystems) to qRT-PCR on ABI PRISM 7300 RT-PCR system (Applied Biosystems). GADPH served as endogenous control gene for the normalization of the gene levels. 2^−ΔΔCt^ method was used to obtain the related quantitative expression of RNA. The specific primers were as follows: MALAT1, (forward) 5-CTCCCCACAAGCAACTTCTC-3 and (reverse) 5-TTCAACCCACCAAAGACCTC-3; Emr1, (forward) 5-TTTTCAGATCCTTGGCCATC-3 and (reverse) 5-GGGTGGCAAGTGCAGAAGTA-3; CD68, (forward) 5-TGTTCAGCTCCAAGCCCAAA-3 and (reverse) 5-GTACCGTCACAACCTCCCTG-3; IL-1β, (forward) 5-TTCATCTTTGAAGAAGAGCCCAT-3 and (reverse) 5-TCGGAGCCTGTAGTGCAGTT-3; IL-6, (forward) 5-TCCAGTTGCCTTCTTGGGAC-3 and (reverse) 5-AGTCTCCTCTCCGGACTTGT-3; TNF-α, (forward) 5-AGCCGATGGGTTGTACCTTG-3 and (reverse) 5-ATAGCAAATCGGCTGACGGT-3; GADPH, (forward) 5-CGGATTTGGTCGTATTGGG-3 and (reverse) 5-CTGGAAGATGGTGATGGGATT-3. The thermal conditions for all PCR reactions were as follows: 95 °C for 10 mins, 40 cycles of 95 °C for 15 s, 60 °C for 45 s.

### Western blot assay

Western blot assay was performed as described in our previous study^20^. Briefly, the protein extracts were heated with the sample buffer for 10 mins, followed by divided on a 10% polyacrylamide gel, and then transferred into the PVDF membrane. The membranes were blocked with 5% BSA, followed by maintained with primary antibodies for MyD88 (1:1000, Cell Signaling Technology), IRAK1 (1:2000, Cell Signaling Technology) and TRAF6 (1:1000, Santa Cruz Biotechnology). In addition, antibodies against IL-1β (1:2000, Santa Cruz Biotechnology), IL-6 (1:1000, Cell Signaling Technology) and TNF-α (1:2000, Santa Cruz Biotechnology) were used. Then the membranes were rinsed in TBST to be incubated with corresponding secondary antibodies at room temperature for 1 h. β-actin was used to act as a loading control. LI-COR Odyssey System was used to vision the protein bands in the membranes.

### Cell culture and treatment

Primary microglia were isolated from the CNS tissue of neonatal rats as described in our previous study^20^. Rat immortalized microglia cell line (HAPI cells) were re-suspended in Dulbecco’s modified Eagle’s medium (DMEM) containing 10% fetal bovine serum (FBS) at 37 °C in 5% CO_2_. The medium with 30 mM glucose and 1% FBS was used to simulate diabetes environment for cells, and the medium containing normal glucose (5.5 mM) was used as a negative control. To establish hypoxia/reoxygenation (H/R) injury cell model, HAPI cells in glucose-free culture medium were cultured at 37 °C with 5% CO_2_, 1% O_2_ and 94% N_2_ for 4 h, and then cultured at 37 °C with 5% CO_2_, 21% O_2_ and 74% N_2_ for 2 h in full culture medium. In the following experiments, HAPI cells were transfected with si-MALAT1 using siRNA transfection reagent, followed by treated with high glucose and H/R.

For transfection experiment, HAPI cells (2 × 10^5^ cells/well) were cultured in 24-well plates overnight and then transfected with siRNA-MALAT1 or siRNA-MyD88 (1 μg) using siRNA transfection reagent (4 μL), or transfected with pcDNA-MALAT1 using Lipofectamine 2000 (Invitrogen) according to the manufacturer’s instructions. The siRNA-MALAT1 (5-GACAGGTATCTCTTCGTTATC-3) and siRNA-MyD88 (5-GAUGAUUACCUGCAGAGCA-3) sequences were designed and synthesized by Biowit Technologies (Shenzhen, China). The overexpressing plasmid of MALAT1 (pcDNA-MALAT1) was synthetized by Shanghai GenePharma Co., Ltd (Shanghai, China). After 48 h of transfection, the gene expression and protein expression was determined by qRT-PCR and western blot, respectively.

### Chromatin immunoprecipitation (ChIP) assay

Approximately 1 × 10^6^ HAPI cells were cross-linked with a 1% final concentration of formaldehyde (Sigma-Aldrich) at 37 °C for 10 mins. ChIP assay was performed with the commercial kit (Beyotime, China) in accordance to the manufacturer’s protocol. The antibodies acetyl-histone H3/H4 or and control normal IgG were purchased from Santa Cruz Biotecnology. ChIP-purified DNA was amplified by standard PCR using primers specific for the MyD88 promoter and the 2 × PCR Master Mix (Promega). After PCR reaction, PCR products were separated on 1.2% agarose gels and visualized by Gel imaging system software (Tanon, Shanghai). Specific enrichment is calculated using the cycle threshold (Ct): 2^(Ct of control ChIP–Ct of control Input)^/2^(Ct of AcH3/H4 ChIP–Ct of AcH3/H4 Input)^.

### Cell proliferation and apoptosis assays

To assess the survival of HAPI cells, MTT assay were performed. HAPI cells (1 × 10^4^ cells/well) were cultured into 96-well plates under corresponding conditions, and then MTT (5 mg/mL, 20 μL) was added into each well and incubated for 4 h at 37 °C. The supernatant was then aspirated, and added with dimethylsulfoxide (DMSO) (150 μL) to agitation for 10 mins to dissolve crystals at room temperature. The absorbance values were surveyed using an ELISA reader (Bio-Rad Laboratories, Richmond, CA) at 490 nm.

Cells that need to be performed with apoptosis assay were collected by centrifugation at 1,500 × g for 3 mins, and washed twice with ice-cold phosphate buffered solution (PBS) and then colourred by the Annexin V-FITC Apoptosis Detection Kit (BD, San Jose, USA) in accordance with the manufacturer’s instructions. The cells were incubated with Annexin V-FITC (5 μL) and PI (5 μL) for 15 mins in Annexin V-FITC binding buffer in the dark at room temperature. Cells were then assessed by using a flow cytometer (BD FACS Calibur, Becton, Dickinson and Company Biosciences, San Jose, USA). Each experiment was repeated three times.

### Statistical analysis

For the statistical analysis, SPSS 16.0 software for Windows was used to survey. All the data were indicated as means ± standard deviations. A single comparison between two groups was surveyed by Student’s t test, and one-way ANOVA with Bonferroni post-hoc test was used for multiple comparison. P < 0.01 was considered statistically significant for the differences.

## Results

### DM exacerbated cerebral I/R injury

**Figure 1:**
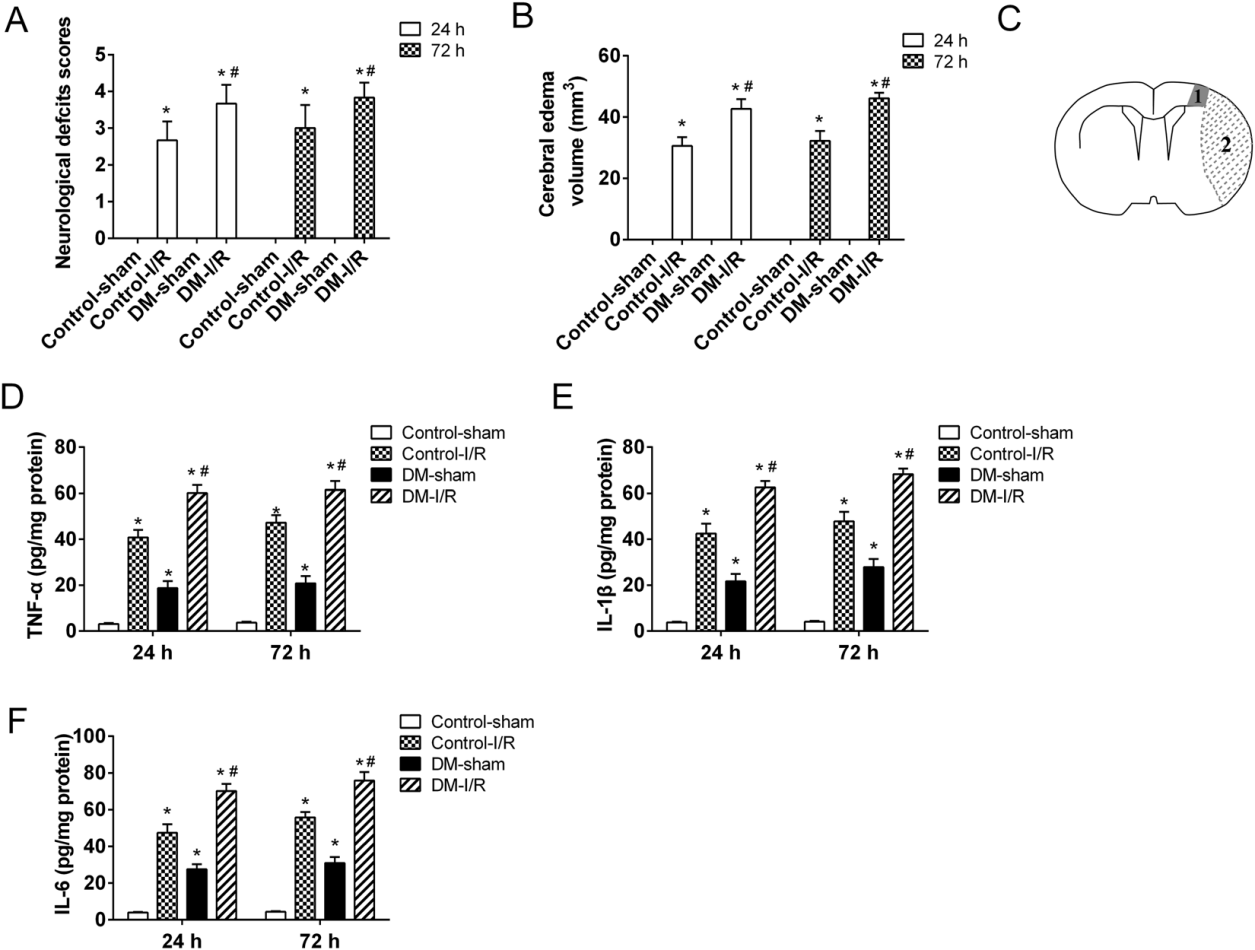
Establishment of cerebral ischemic reperfusion (I/R) injury model in diabetic rats. SD rats were randomly divided into 4 groups: Control-sham, Control-I/R, DM-sham, DM-I/R. The diabetic rats (n = 12) were induced by intraperitoneal injection of streptozotocin (STZ, 60 mg/kg). The another 12 rats were received the equal volume of citrate buffer. The cerebral I/R injury models in diabetic rats (DM-I/R, n = 6) were established by middle cerebral artery occlusion (MCAO). The diabetic rats were sham operated, as control for cerebral I/R injury (DM-sham, n = 6). The rats with citrate buffer received MCAO (Control-I/R, n = 6) and sham operation (Control-sham, n = 6), respectively. **(A)** The functional neurological deficit score was assessed and **(B)** the brain edema was determined at 24 and 72 hours after cerebral I/R injury. **(C)** A schematic of the peri-infarct area. 1: peri-infract cortex; 2: ischemic core. The secretion levels of **(D)** TNF-α, **(E)** IL-1β and **(F)** IL-6 in the peri-infarct cortical tissues were detected by ELISA assay. *P < 0.01 vs. Control-sham; ^#^P < 0.01 vs. Control-I/R.

The neurological deficits in the rats with cerebral I/R injury and/or DM were assessed using Longa 5 scores. Results showed that the rats (n = 6) with cerebral I/R injury and DM (DM-I/R) had a significantly higher neurological deficit scores compared with the control (n = 6) and cerebral I/R injury rats (n = 6) both at 24 and 72 hours after injury (Fig. 1A). Moreover, the cerebral edema volume in DM-I/R rats (n = 6) was significantly larger than the control (n = 6) and cerebral I/R injury rats (n = 6) (Fig. 1B). HG-induced inflammatory response was confirmed to implicate in the pathogenesis of various complications of DM, such as diabetic retinopathy (DR) and diabetic nephropathy (DN). In the present study, it was observed that the expressions of the pro-inflammatory cytokines TNF-α, IL-1β and IL-6 were dramatically increased in the peri-infarct cortical tissues of DM-I/R rats (n = 6) compared with the control (n = 6) and cerebral I/R injury rats (n = 6) (Fig. 1C–F). These findings suggested that DM exacerbated cerebral I/R injury accompanied by inflammatory response.

### The expression profile of MALAT1, MyD88, IRAK1 and TRAF6 in DM-I/R

**Figure 2:**
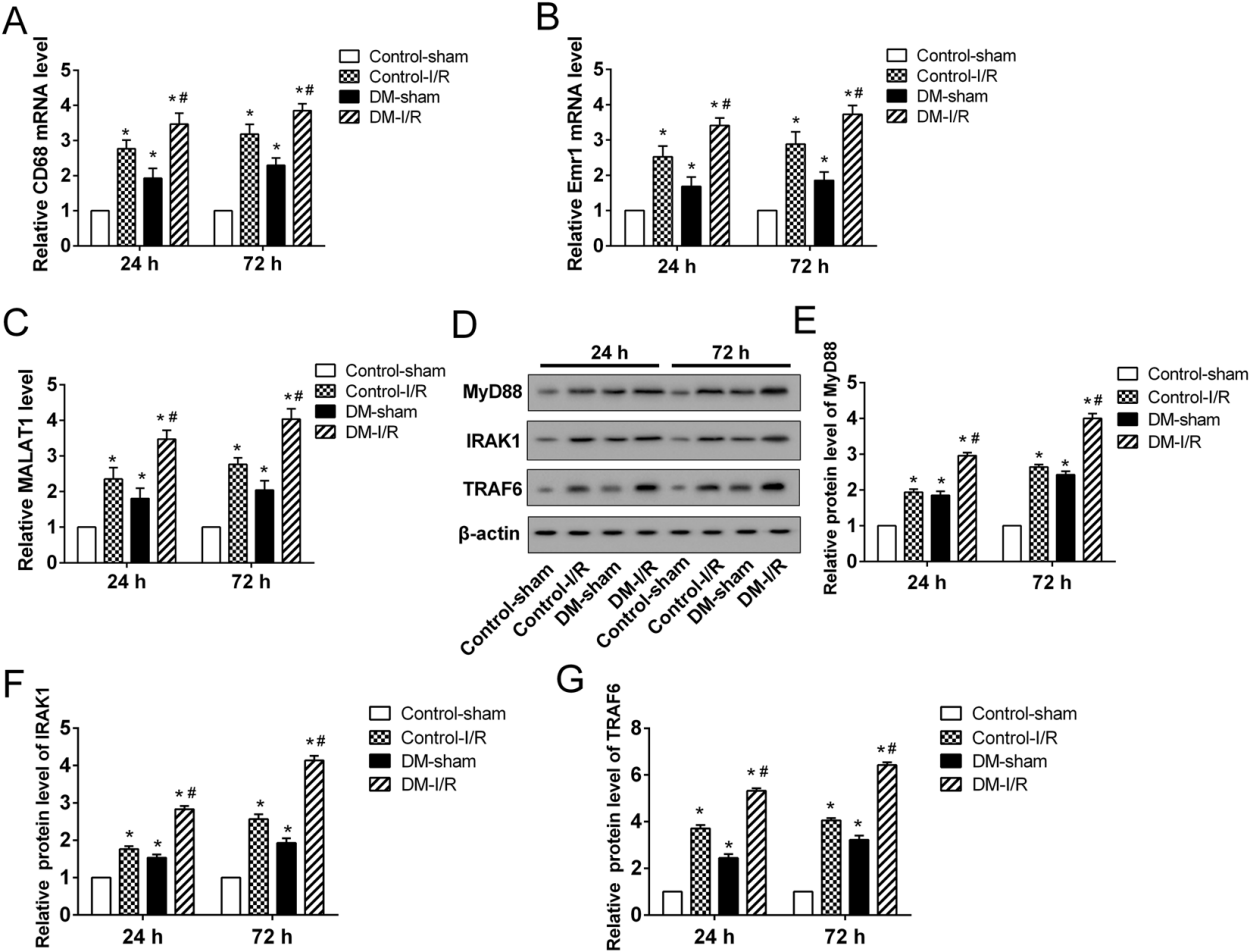
The expression of MALAT1, MyD88, IRAK1 and TRAF6 in DM-I/R rats at 24 and 72 hours after cerebral I/R injury. The mRNA expressions of **(A)** CD68 and **(B)** Emr1, which were the makers of the microglial cells in the peri-infarct cortical tissue of rats, were determined by qRT-PCR. **(C)** The expression of MALAT1. **(D)** Representative Western blot analysis of MyD88, IRAK1, TRAF6 protein. The relative expressions of **(E)** MyD88, **(F)** IRAK1, **(G)** TRAF6 protein were measured. *P < 0.01 vs. Control-sham; ^#^P < 0.01 vs. Control-I/R.

**Figure 3:**
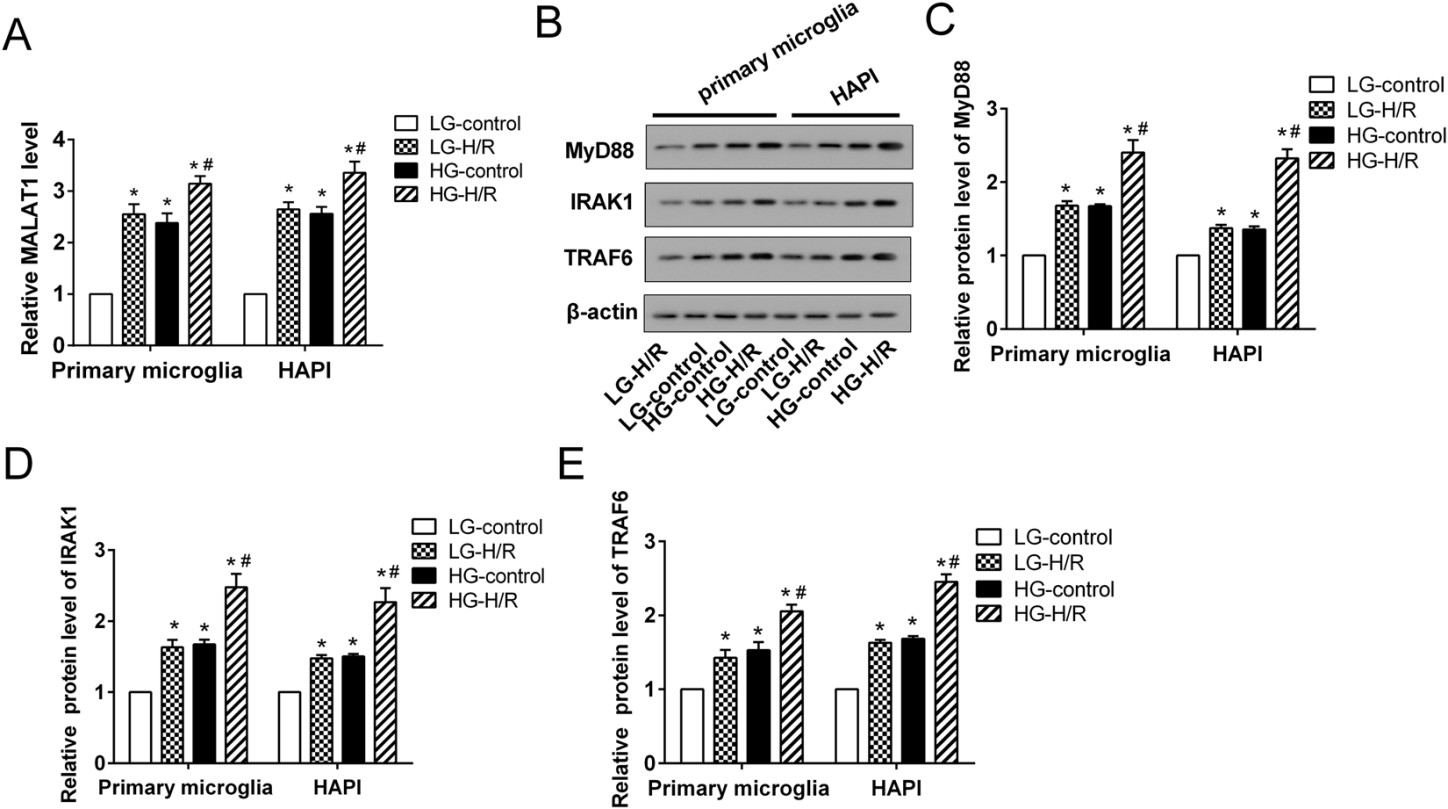
The expression of MALAT1, MyD88, IRAK1 and TRAF6 in an *in vitro* model of DM-I/R. The rat primary microglia and microglia line HAPI were cultured to establish the cell model of DM-I/R with high glucose (HG) and hypoxia-reoxygenation (H/R). The cells were divided into 4 groups: LG-control (low glucose treatment, 5.5 mM), LG-H/R (LG and H/R treatment), HG-control (HG treatment, 30 mM), HG-H/R (HG and H/R treatment). **(A)** The expression of MALAT1. **(B)** Representative Western blot analysis of MyD88, IRAK1, TRAF6 protein. The relative expressions of **(C)** MyD88, **(D)** IRAK1, **(E)** TRAF6 protein were measured. ^*^P < 0.01 vs. LG-control; ^#^P < 0.01 vs. LG-H/R.

Over-activated microglial cells play critical roles in the pro-inflammatory response of the CNS via the increased release of pro-inflammatory cytokines. The mRNA expressions of CD68 and Emr1, the makers of microglial cells, were dramatically increased in the peri-infarct cortical tissues of cerebral I/R injury rats (n = 6), DM rats (n = 6) and DM-I/R rats (n = 6) compared with the control (n = 6) (Fig. 2A,B). Besides, the relative expressions of CD68 and Emr1 in peri-infarct cortical tissues of DM-I/R rats were significantly higher than that of cerebral I/R injury rats, suggesting that DM enhanced the activation of microglial cells (Fig. 2A,B). Moreover, the expression of MALAT1 was significantly increased in the peri-infarct cortical tissues of cerebral I/R injury rats (n = 6), DM rats (n = 6) and DM-I/R rats (n = 6) compared with the control (n = 6), of which MALAT1 increased most dramatically in DM-I/R rats (Fig. 2C). It was also observed that the protein expressions of MyD88, IRAK1 and TRAF6 were significantly up-regulated in the peri-infarct cortical tissues of cerebral I/R injury rats (n = 6), DM rats (n = 6) and DM-I/R rats (n = 6) (Fig. 2D–G). This observation was further confirmed by the results of the *in vitro* models of DM-I/R. The rat primary microglia cells and microglia line HAPI were cultured to establish the cell model of DM-I/R by treatment of HG and H/R. Compared with the LG-control, the expression of MALAT1 in microglia was significantly induced by H/R and/or HG, as well as MyD88, IRAK1 and TRAF6 protein (Fig. 3A–E). Compared with the LG-H/R, H/R combined with HG induced a higher expression of MALAT1, MyD88, IRAK1 and TRAF6 protein in both primary microglia cells and HAPI cells (Fig. 3A–E).

### MALAT1 promoted the HG-H/R-induced inflammatory response in microglia

**Figure 4:**
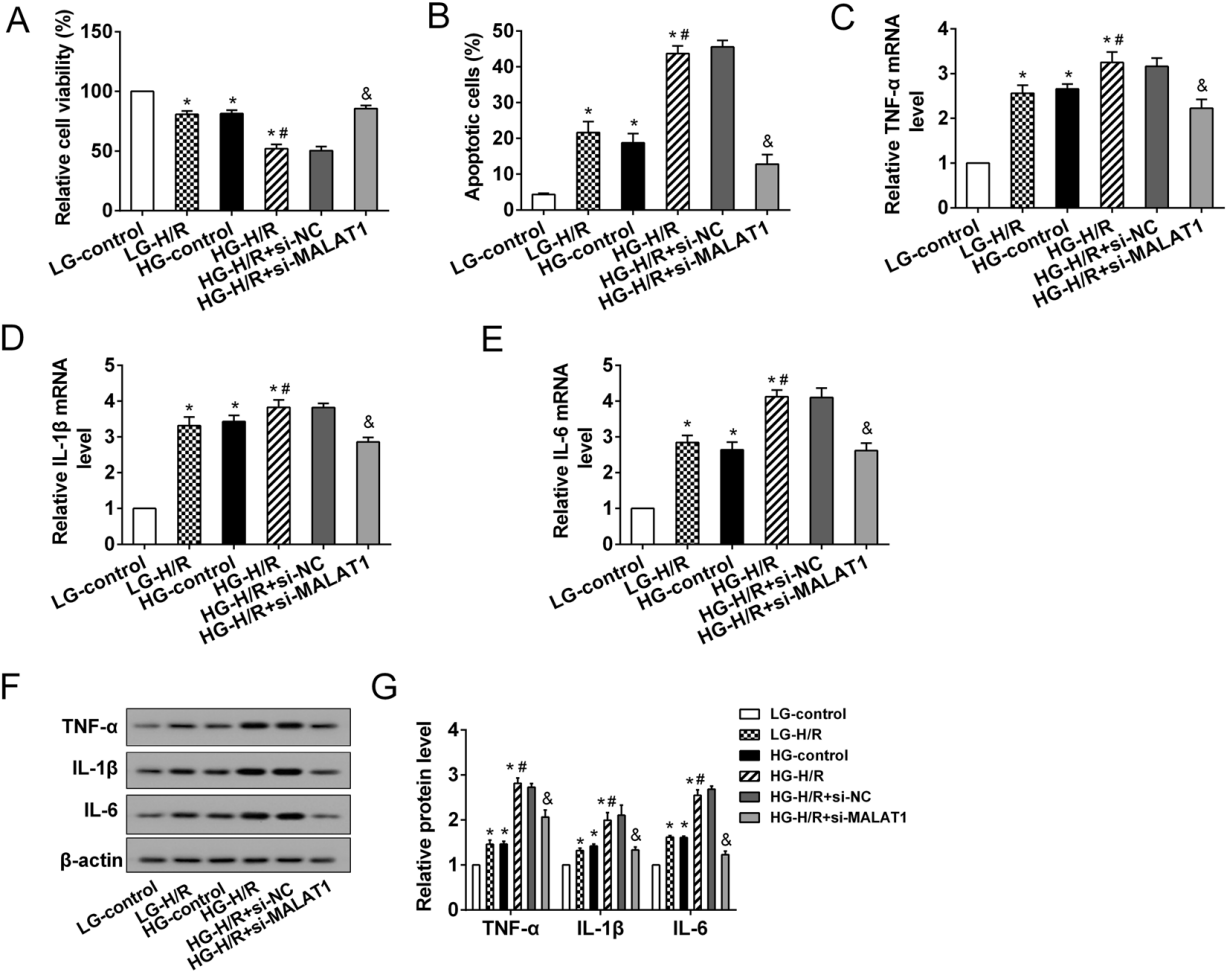
The effects of MALAT1 on microglia *in vitro*. The HAPI cells were divided into 6 groups: LG-control, LG-H/R, HG-control, HG-H/R, HG-H/R + si-NC, HG-H/R + si-MALAT1. **(A)** The relative cell viability was detected with MTT assays. **(B)** The apoptotic cell was assessed by flow cytometry. **(C–E)** The expression of proinflammatory cytokines (TNF-α, IL-1β and IL-6) was measured at mRNA levels. **(F)** Representative Western blot analysis of TNF-α, IL-1β and IL-6. **(G)** The relative expressions of TNF-α, IL-1β, and IL-6 protein were measured. *P < 0.01 vs. LG-control; ^#^P < 0.01 vs. LG-H/R; ^&^P < 0.01vs. HG-H/R + si-NC.

To determine the functional role of MALAT1 in the HG-H/R-induced inflammatory response of microglia, MALAT1 was inhibited in HAPI cells using transfection of siRNA targeting MALAT1 (si-MALAT1). The results showed that the relative cell viability of HAPI cells was significantly reduced by pretreatment of H/R, HG or HG-H/R, which was accompanied by a substantially increase in apoptosis (Fig. 4A,B). We found that MALAT1 silencing enhanced cell viability and inhibited cell apoptosis in HAPI cells treated with HG-H/R (Fig. 4A,B). HG and H/R alone or together evidently induced the expression of proinflammatory cytokines TNF-α, IL-1β and IL-6 at both mRNA and protein levels in HAPI cells (Fig. 4C–G). As expected, MALAT1 silencing could markedly attenuate the effects of HG-H/R on HAPI cells, as seen by a decrease in proinflammatory cytokines expression (Fig. 4C–G). These results suggested that MALAT1 played a vital role in the HG-H/R-induced inflammatory response of microglia.

### MALAT1 positively regulated the expression of MyD88, IRAK1 and TRAF6 protein in microglia

**Figure 5:**
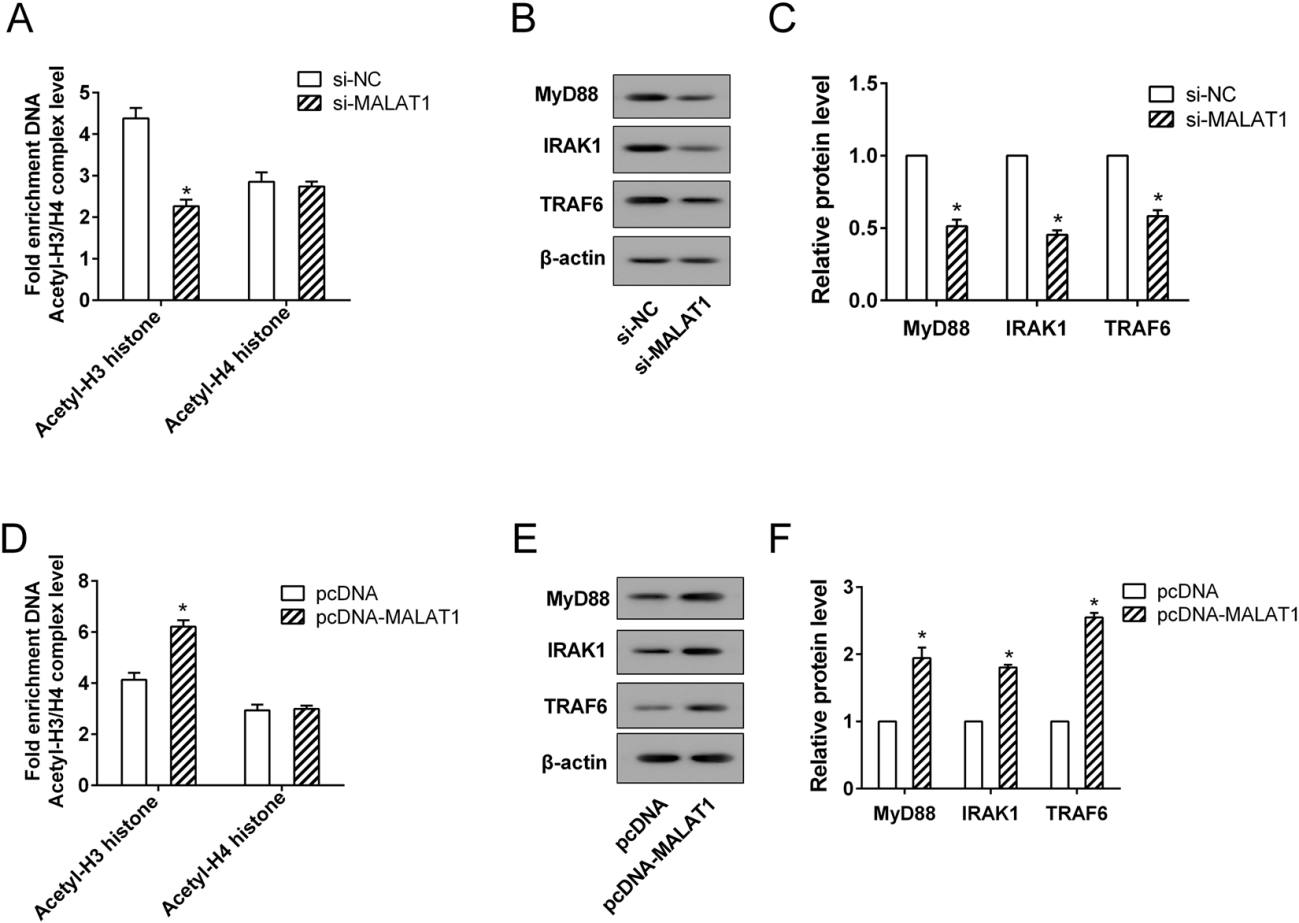
The regulation of MALAT1 on MyD88/IRAK1/TRAF6 axis. The HAPI cells were transfected with si-MALAT1, pcDNA-MALAT1, si-NC and pcDNA, respectively. **(A**,**D)** The levels of H3 and H4 histone acetylation at the promoter of MyD88 were assessed and **(B**,**C**,**E**,**F)** the protein expressions of MyD88, IRAK1 and TRAF6 were measured. *P < 0.01 vs. si-NC or pcDNA.

To further study the mechanism of MALAT1, we explored the functional relationship between MALAT1 and MyD88. Our results showed that the level of H3 histone acetylation at the promoter of MyD88 was down-regulated by si-MALAT1, but increased by pcDNA-MALAT1 (Fig. 5A,D). However, MALAT1 had no significant effect on H4 histone acetylation of MyD88 promoter (Fig. 5A,D). Furthermore, the protein expressions of MyD88, IRAK1 and TRAF6 were significantly reduced in HAPI cells with MALAT1 silencing (Fig. 5B,C); while MALAT1 overexpression obviously enhanced the protein expressions of MyD88, IRAK1 and TRAF6 (Fig. 5E,F). These findings indicated that MALAT1 positively regulated the expression of MyD88 via increasing the level of H3 histone acetylation of MyD88 promoter, thereby affecting the expression of MyD88-regulated proteins, IRAK1 and TRAF6.

### MALAT1 induced the inflammatory response by MyD88 in the HG-H/R treated microglia

**Figure 6:**
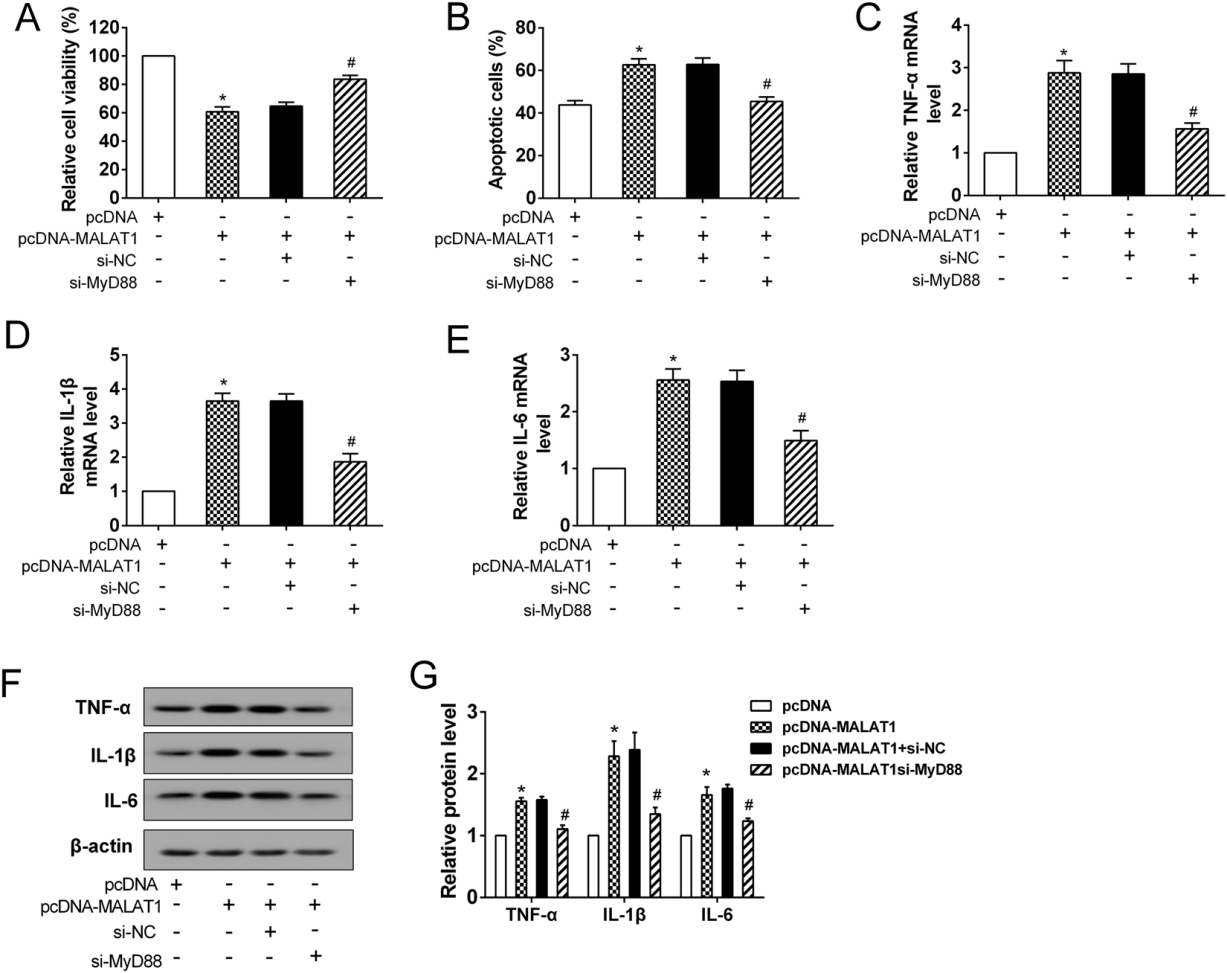
MyD88 was a downstream molecule of MALAT1 in microglia. The HAPI cells were divided into 4 groups: pcDNA, pcDNA-MALAT1, pcDNA-MALAT1 + si-NC, pcDNA-MALAT1 + si-MyD88. After transfection, all cells were treated with HG-H/R. **(A)** The relative cell viability and **(B)** apoptosis were assessed. **(C–E)** The expression of proinflammatory cytokines (TNF-α, IL-1β and IL-6) was measured at mRNA levels. **(F)** Representative Western blot analysis of TNF-α, IL-1β and IL-6. **(G)** The relative expressions of TNF-α, IL-1β, and IL-6 protein were measured. *P < 0.01 vs. pcDNA; ^#^P < 0.01 vs. pcDNA-MALAT1 + si-NC.

MALAT1 overexpression induced the growth inhibition of HAPI cells by decreasing cell viability and promoting apoptosis, but MyD88 silencing significantly attenuated the effects of MALAT1 on the growth of HAPI cells (Fig. 6A,B). MALAT1 overexpression dramatically accelerated the expression of proinflammatory cytokines TNF-α, IL-1β and IL-6 at both mRNA and protein levels in the HG-H/R treated HAPI cells; while the up-regulation was attenuated considerably by MyD88 knockdown (Fig. 6C–G). Collectively, our data suggested that MyD88 was crucial for the MALAT1-induced inflammatory response in microglia.

## Discussion

It has been showed that diabetes-associated hyperglycemia increased ischemic infarct volumes and was closely correlated with the poor prognosis of stroke^21^. The excessive inflammation response facilitated the cranial nerves injuries following cerebral ischemic reperfusion (I/R)^22^. In the current study, the cerebral I/R injury model in diabetic rats had more severe neuronal injury both at 24 and 72 hours after I/R was set. Moreover, the levels of the proinflammatory cytokines TNF-α, IL-1β and IL-6 were dramatically increased in the peri-infarct cortical tissues of diabetic rats with cerebral I/R injury. Consistent with of previous studies, we specified that DM exacerbated cerebral I/R injury accompanied by increasing inflammatory response of microglia *in vivo* and *in vitro*.

Microglia activation and release of inflammatory factors are central events in inflammatory response^23^. Microglia is the main inflammatory cell that participates in the pathological environment of cerebral I/R injury, which can secrete a large amounts of inflammatory cytokines, resulting in serious inflammation reaction^14^. Hyperglycemia has been found to aggravate neuronal degeneration, apoptosis, and inflammation in ischemic regions^24^. It also has been proved that hyperglycemia promoted microglia activation, thereby further worsening ischemic brain injury^25^. The present study identified activated microglia and a large number of inflammatory cytokines in the peri-infarct cortical tissues during cerebral I/R injury associated with DM. We also observed that the expression of MALAT1 was significantly increased in DM-I/R models when compared with I/R models, suggesting that MALAT1 played a critical role in the pathogenesis of DM-associated cerebral I/R injury. MALAT1 was closely related to inflammatory reaction in a variety of pathological and physiological circumstances, including DR and diabetes-induced vascular complications. Our results confirmed that MALAT1 promoted the HG-H/R-induced inflammatory response in microglia, suggesting that MALAT1 might play an important role in the progression of DM-associated cerebral I/R injury.

After confirming the important role of MALAT1 in the inflammatory response during cerebral I/R injury associated with DM, we also identified the downstream signaling pathway of MALAT1 in this mechanism. The protein expressions of MyD88, IRAK1 and TRAF6 were found to up-regulate in the peri-infarct cortical tissue of I/R and DM-I/R models and this observation was further confirmed by the findings of the *in vitro* model of DM-I/R. MyD88-dependent signaling was the essential pathway for provoking the systemic inflammatory reaction and NF-κB activation in the process of cerebral I/R injury^26^. The MyD88 adaptor proteins, TRAF6 and IRAK1, could assemble into a complex that induced the activation of NF-κB cascade reaction^27^. In this study, we identified that MALAT1 up-regulated the expression of MyD88 through increasing the H3 histone acetylation of MyD88 promoter, thereby increasing IRAK1 and TRAF6 protein. Additional studies are necessary to delineate how MALAT1 affects the H3 histone acetylation of MyD88 promoter.

**Figure 7:**
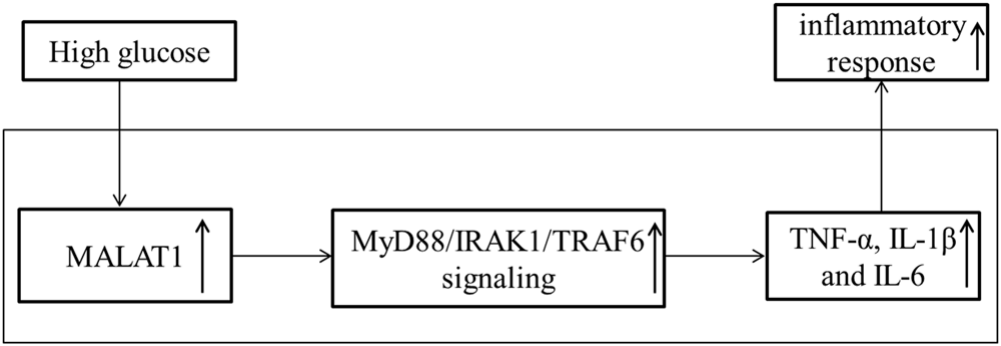
The pictorial representation of MALAT1 correlation with high glucose-induced inflammatory response of microglial cells.

In the present study, the cerebral I/R injury was aggravated by DM in the rat models. We further demonstrated that MALAT1 triggered the inflammatory response in microglia via MyD88 signaling that mediated the DM-induced exacerbation of cerebral I/R injury (Fig. 7). MALAT1 is apparently an important regulating factor in DM-associated cerebral I/R injury, and it also may be the effective therapeutic target for preventing and combating DM-associated cerebral I/R injury.
